# Supplementary material for: Predicting visual function by interpreting a neuronal wiring diagram
Source: Nature. 2024 Oct 2;634(8032):113–23. doi: 10.1038/s41586-024-07953-5 (PMC11446822; doi:10.1038/s41586-024-07953-5)
Supplement: Supplementary file 6 — CRF and ERF predictions for individual TmY4 and TmY9 cells. Analogous to Supplementary Data 3, but for TmY target types. Shown are the top four monosynaptic pathways, the strongest pathway passing through each of the top ten intermediary types (ranking from Extended Data Fig. 7), and the trisynaptic pathway Tm1–TmY–Dm3–TmY (see the section entitled Prediction of spatial normalization). [file 41586_2024_7953_MOESM6_ESM.zip › DataS4/TmY4/720575940646534020.pdf]

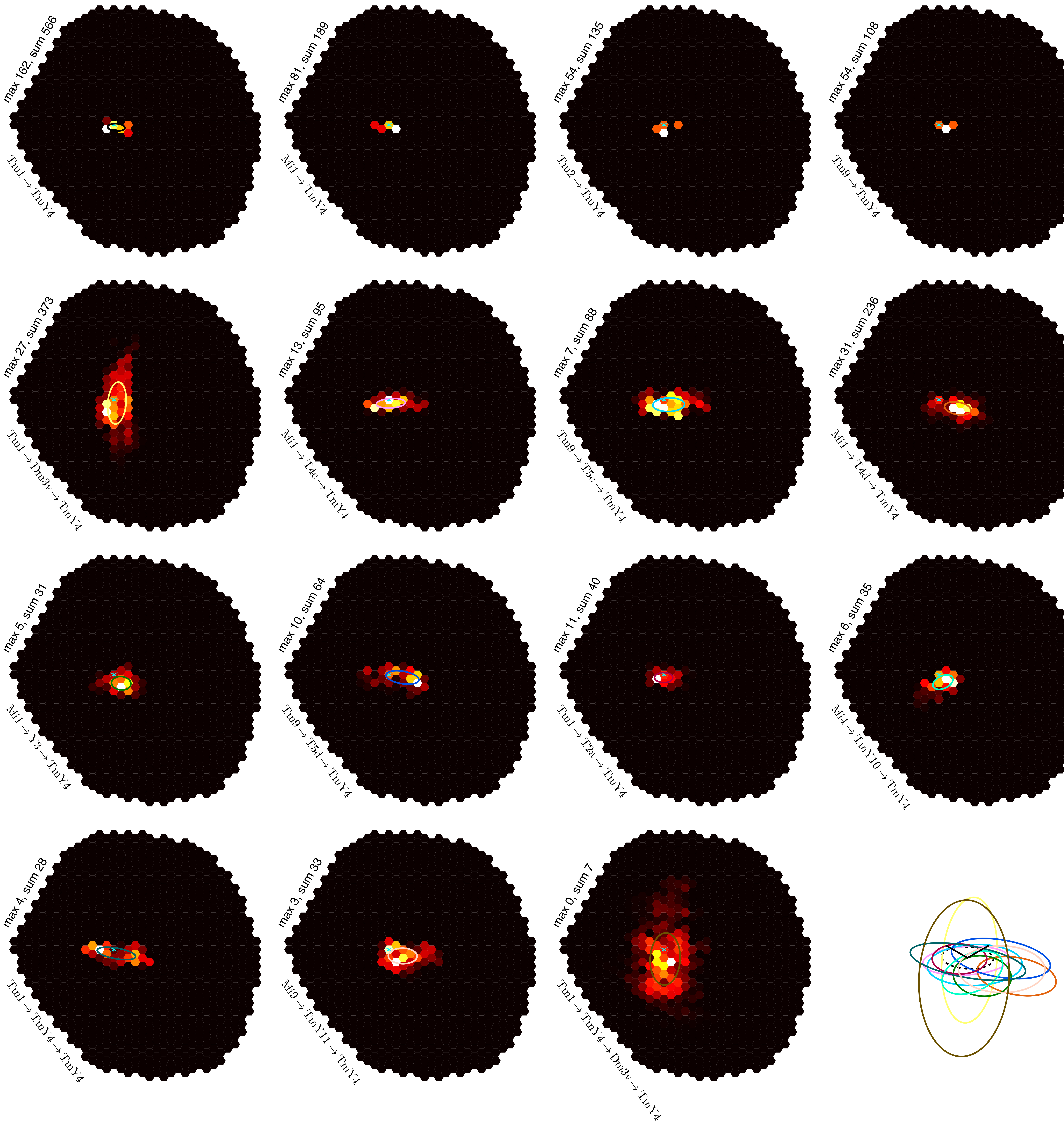
max 162, sum 566
Tm1 → TmY4
max 81, sum 189
Mi1 → TmY4
max 54, sum 135
Tm2 → TmY4
max 54, sum 108
Tm9 → TmY4
max 27, sum 373
Tm1 → Dm3v → TmY4
max 13, sum 95
Mi1 → T4c → TmY4
max 7, sum 88
Tm9 → T5c → TmY4
max 31, sum 236
Mi1 → T4d → TmY4
max 5, sum 31
Mi1 → Y3 → TmY4
max 10, sum 64
Tm9 → T5d → TmY4
max 11, sum 40
Tm1 → T2a → TmY4
max 6, sum 35
Mi4 → TmY10 → TmY4
max 4, sum 28
Tm1 → TmY4 → TmY4
max 3, sum 33
Mi9 → TmY11 → TmY4
max 0, sum 7
Tm1 → TmY4 → Dm3v → TmY4
